# Institutional Questions

**Published:** 1900-01-20

**Authors:** 


### INSTITUTIONAL QUESTIONS.

### Temporary or Permanent Hospital Ships.

"HOSPITAL" asks: I have seen it suggested that hospital ships for military purposes should be fitted up and kept always in readiness for such emergencies as the present. Has not, however, experience shown it to be undesirable to adopt such a course as this, and is it not better to pursue the plan now followed of chartering the best available ships as occasion arises, and having these adapted to hospital requirements on the most approved lines?

*** Probably the latter system is the best, and that for several reasons. Under no circumstances can a ship be an ideal place for the reception of the sick, but no ships built can be better adapted to such a purpose than the splendid vessels belonging to the various passenger steamship companies. The most modern appliances can then be used, and the newest inventions applied. If, on the other hand, hospital ships were built at great cost, and fitted up for that special purpose, the expense of renovating their arrangements whenever, sometimes at long intervals, they were needed for service would be great, and would probably not be incurred. The sick would therefore suffer more than they do at present in transport.

### Hong Kong Hospitals.

"ORIENTAL" asks: Are there any other hospitals in Hong Kong for Europeans but the Government Civil Hospital, and what number of beds does the latter institution contain?

*** We do not know of any private hospitals for Europeans in Hong Kong. The Government Civil Hospital has accommodation for 132 beds. The London Missionary Society has two hospitals, the Alice Memorial Hospital and the Nethersole, the former with fifty-three beds, the latter with forty, but these are for native patients.

### Fever Hospitals.

"DISTRICT COUNCILLOR" writes: Can you kindly direct me to information concerning the construction and general arrangement of isolation hospitals, with details respecting fittings, cost, plans, &c.?

*** If you can obtain access to Sir Henry Burdett's "Hospitals and Asylums of the World" you will there find much information of the kind you require. The same writer's volume on "Cottage Hospitals" (Scientific Press, 28, Southampton Street, Strand, W.C.) also gives descriptions of smaller isolation hospitals; and another book which might be valuable would be Dr. McNeill's "Prevention of Epidemics, and Construction and Management of Isolation Hospitals" (J and A. Churchill, 11, New Burlington Street).

